# Moving On in Vietnam

**DOI:** 10.1289/ehp.114-a156

**Published:** 2006-03

**Authors:** Luz Claudio

War and unrest mark the history of Vietnam. For hundreds of years, Vietnam has fought a number of foreign invaders including the French, Japanese, and Chinese. The U.S.–Vietnam War was just one of several chapters in this history, ending in 1973 with the Paris Peace Accords. The American War, as it is referred to in Vietnam, has been estimated to have resulted in the deaths of 1.5 million Vietnamese and 58,000 Americans. The countryside is still dotted by unexploded mines, and questions remain about environmental health concerns such as the lasting effects of dioxin-containing herbicides used during the war.

But as a bumper sticker spotted on a Hanoi street pointed out, “Vietnam is a country, not a war,” and today many of Vietnam’s most pressing concerns are related to post-war growth. About half of Vietnam’s current population of 80 million people is under 30 years old. This young generation, many of whom have a limited recollection of wartime, drives a sense of optimism about the future of the country. Foreign investment in this country for 2005 is estimated at US$5.4 billion, only a little less than investment in India, and Vietnam is expected to enter the World Trade Organization in mid-2006. This growth makes Vietnam a duality of a country, on the one hand facing the environmental problems typical of the rural countryside (such as those related to poor sanitation), and on the other facing the occupational and environmental health consequences of rapid industrialization.

This was the setting of the joint Second International Scientific Conference on Occupational and Environmental Health and Sixth National Scientific Conference on Occupational Health, held in Hanoi on 16–18 November 2005. The conference gathered 176 participants with 194 abstract presentations. Most of the participants were from Vietnam; 44 were from abroad, mostly from other Asian countries. The conference was organized by the Vietnam Association of Occupational Health and the University of Washington, with the sponsorship of numerous Vietnamese, international, and U.S. agencies, including the NIEHS, the National Institute for Occupational Safety and Health (NIOSH), and the John E. Fogarty International Center of the NIH.

Early in the conference NIEHS director David Schwartz and Anne Sassaman, director of the NIEHS Division of Extramural Research and Training, presented the vision of the NIEHS for expanding global health as part of its five-year strategic plan and bringing the institute’s expertise to bear on the problems facing this growing country. “NIEHS-supported research has shown that air pollution, which is a big problem in Vietnam, can impact cardiovascular health and increase susceptibility to respiratory infections,” said Sassaman. “These and other environmental health concerns are a growing problem in the developing world.”

## Powering Up

The United States first established diplomatic relations with Vietnam in 1995, and a bilateral trade agreement signed by the two countries in 2000 opened the door to increasing economic and industrial development in Vietnam. Economic agreements have continued to strengthen with President Bush’s plan to participate in the Asia-Pacific Economic Cooperation Summit to be hosted later this year in Hanoi. In addition to a new wave of U.S. investors, Taiwanese and Japanese business ventures are eager to tap Vietnam’s wealth of cheap, relatively well-educated workers, who are willing to work six days per week, as opposed to the Chinese, who adhere to a five-day work week. Workers in traditional trade villages earn US$60 per month, sometimes less, as the national minimum wage is US$38 per month. (By comparison, the average rate in China is US$90 per month.) Because of these low wages and the relatively new implementation of occupational health standards, Vietnam may be poised to compete with China in the manufacture of goods for export, further expanding environmental and occupational health concerns.

At this point, many multinational companies are adopting a “China plus one” strategy where they continue to support their main manufacturing centers in China and develop subsidiary ventures in another country, increasingly Vietnam. This trend is exemplified in the industrial park located near Hanoi’s international airport. Opened in 2000, the facility already houses 46 factories with more than 16,000 Vietnamese employees. If the Vietnamese government continues to liberalize the economy and improve its infrastructure, the country may significantly increase its share of the global market.

“These investments should include protections for workers in the cities as well as in the rural areas,” said Bui Thanh Tam, who cochaired the session on environmental health and industrial hygiene with conference co-organizer Matthew Keifer, director of the International Scholars in Environmental and Occupational Health program at the University of Washington. With this rapidly expanding industrialization of Vietnam, environmental and occupational health protections and improved scientific capacity in these areas will become increasingly important.

## Health for Workers

The conference therefore focused heavily on occupational health. The establishment of safety codes and means for enforcing occupational and environmental health standards that protect workers and communities was a concern that resonated throughout the conference. A study presented by Ta Quang Buu of the Hai Phong Preventive Medicine Center showed that only 3% of enterprises in the region implemented guidelines for injury prevention. The average number of workdays missed due to occupational injuries was 14.5 per person between the years 2000 and 2004, with 70% of these injuries attributable to violations of occupational safety rules. These data will eventually form the basis of a workplace health promotion program for workers and employers.

However, it is likely that even these figures are underreported. Another presentation described a collaboration between investigators of the Vietnamese National Institute of Occupational and Environmental Health, the Liberty Mutual Research Institute for Safety in the United States, and the University of Massachusetts Lowell that aims to develop a comprehensive injury reporting system in Vietnam. This project will provide information about the scale of the burden of work injuries in the country.

Other presenters described working conditions and the health of workers in the chemical industry. Authors Nguyen The Cong and colleagues at the National Institute of Labour Protection studied data obtained from 24,482 annual medical checkups of workers in this industry. They showed that the most prevalent diseases in this sector were acute ophthalmologic diseases, followed by respiratory and allergic conditions (from 26.8% to 75.1%). There were also findings of silicosis, lead poisoning, and hearing loss among these workers, and the health status of as many as 15.9% of workers was categorized as “unacceptable.” The presenters added that, as Vietnam becomes home to more technical types of ventures that require increased use of industrial chemicals and heavy machinery, these kinds of occupational injuries can be expected to increase unless worker protection guidelines are implemented and enforced.

Much of the manufacturing activity is currently concentrated in traditional trade villages, where families work together to produce one specific commodity, such as pottery, silk garments, furniture, or paper. “Low cost is both a strength and a weakness of this manufacturing enterprise,” said Nguyen Duc Hung of the Institute of Labour Science and Social Affairs in his presentation. In an investigation of six traditional trade villages, Hung found that most of the manufacturing enterprises were located within or in close proximity to the villagers’ living quarters. Most of these settings lacked proper ventilation, chemical disposals, or other protections. Levels of respiratory toxics such as carbon monoxide, sulfur dioxide, and nitrogen dioxide were found to be high in these homes. Hung suggested that labor inspections and regulations that are applied to industrial sites should be expanded to traditional trade villages, where occupational exposures can affect whole families, including children.

## A Need for Sanitation

Despite advances in industrialization, rural and some suburban areas of Vietnam still face a lack of basic sanitation, which affects the availability of safe potable water and causes other environmental health problems. Two studies presented by Le Thi Song Huong and colleagues from the Hai Phong Preventive Medicine Center investigated levels of water contamination with fecal coliforms. Analysis of water samples showed that those with low chlorine residues had high levels of coliforms.

Water sources are likely contaminated by effluent from poorly designed household latrines. These latrines are often located near the house kitchen and have poor drainage. The investigators helped villagers in the An Duong District to build latrines with better drainage and ventilation, located farther from the kitchen, which reduced the contamination of wells and vegetable gardens with fecal microbes. They were able to increase the number of households throughout the area that had these latrines from 19% to 29%. “Rural sanitation is a big concern and a big challenge, and we need implementation of creative new ways to solve these problems,” said Huong.

Another study of fecal contamination of drinking water was conducted in the Cu Jut District in Dac Lac Province by Vuong Tuan Anh and colleagues from the National Institute of Hygiene and Epidemiology of Hanoi. The authors showed that a common source of household drinking water contamination occurred when family members dipped their unwashed hands into water vats stored in the home. The authors speculated that the simple use of long-handled ladles to scoop water combined with hand-washing campaigns could significantly reduce contamination.

## On the Rise

Vietnam, while undeniably on the rise, has far to go to ensure the environmental and occupational health of its citizens. “After visiting Vietnam,” said Schwartz, “it’s abundantly clear that . . . we need to build research capacity in environmental health to effectively address the global health disparities caused by environmental exposures.”

The emergence of international ventures that is permeating Vietnam may yet tip the balance toward a healthier country. Said Le Van Trung, president of the Vietnam Association of Occupational Health and co-organizer of the conference, “This conference is a useful scientific forum and an opportunity for scientists in the local country, in the region, and over the world to exchange information and to discuss occupational and environmental health issues that need to be resolved in the first years of the new millennium.”

## Figures and Tables

**Figure:**
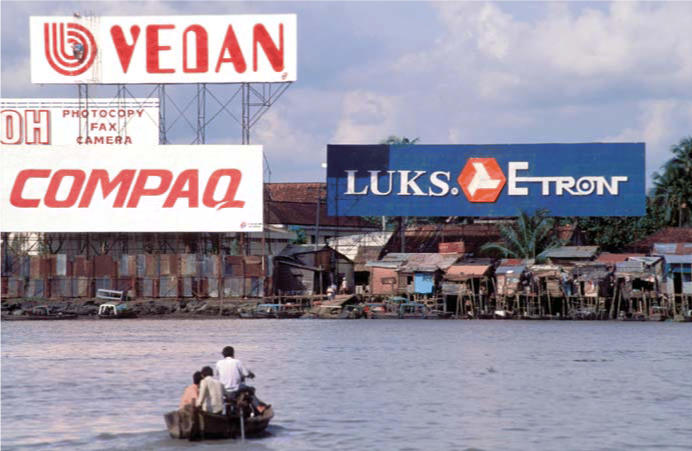
State of flux. Billboards towering over slums along the banks of the Saigon River demonstrate how Vietnam’s infrastructure struggles to keep up with the nation’s rapid industrialization.

**Figure:**
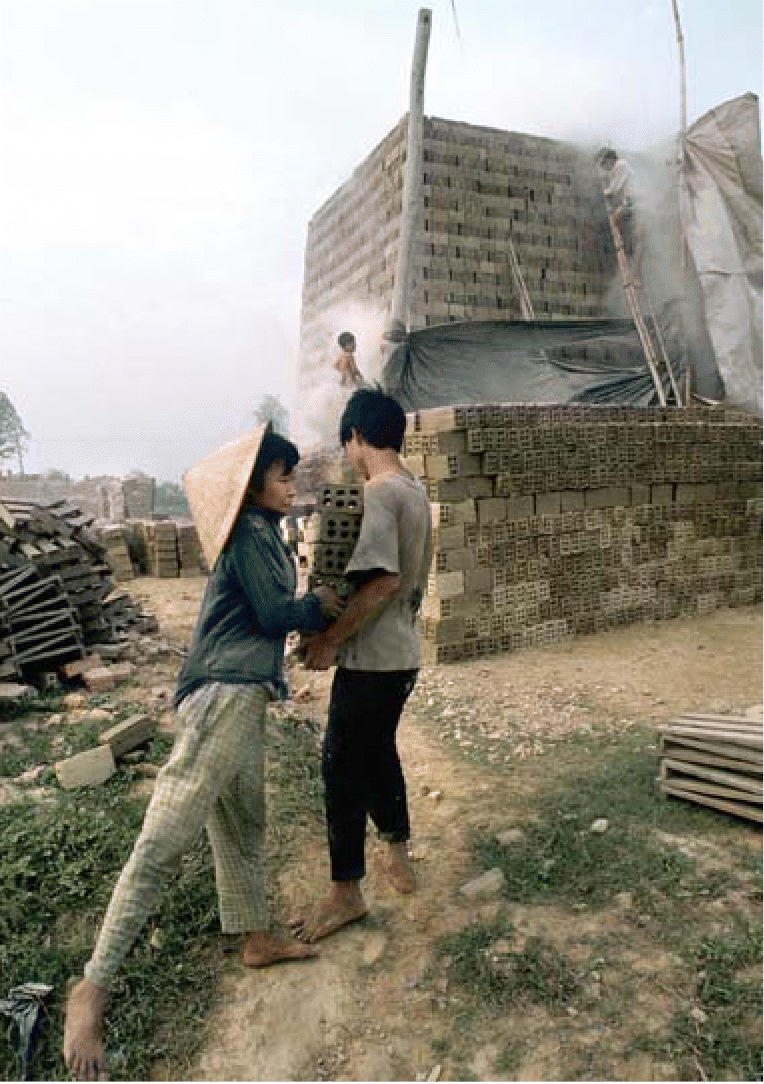
Families at risk. In traditional trade villages, manufacturing enterprises (like this brickyard in Hue Province) and attendant exposures are often located near or in villagers’ homes.

**Figure:**
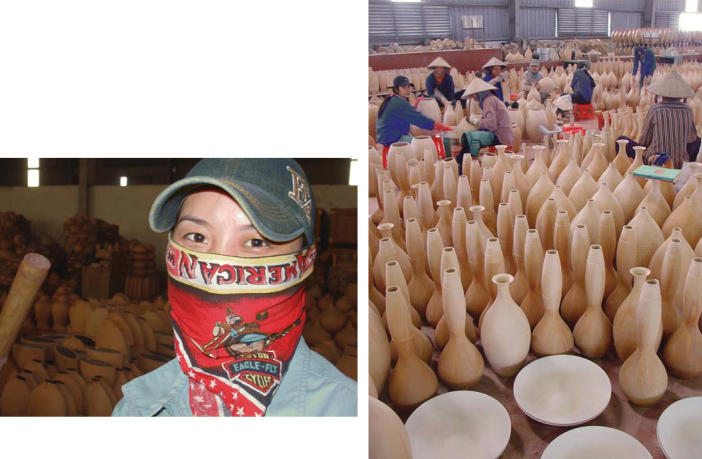
Meeting demand. Improved occupational health standards and safety codes are essential for Vietnam, a country poised to become one of the world’s great manufacturing centers.

